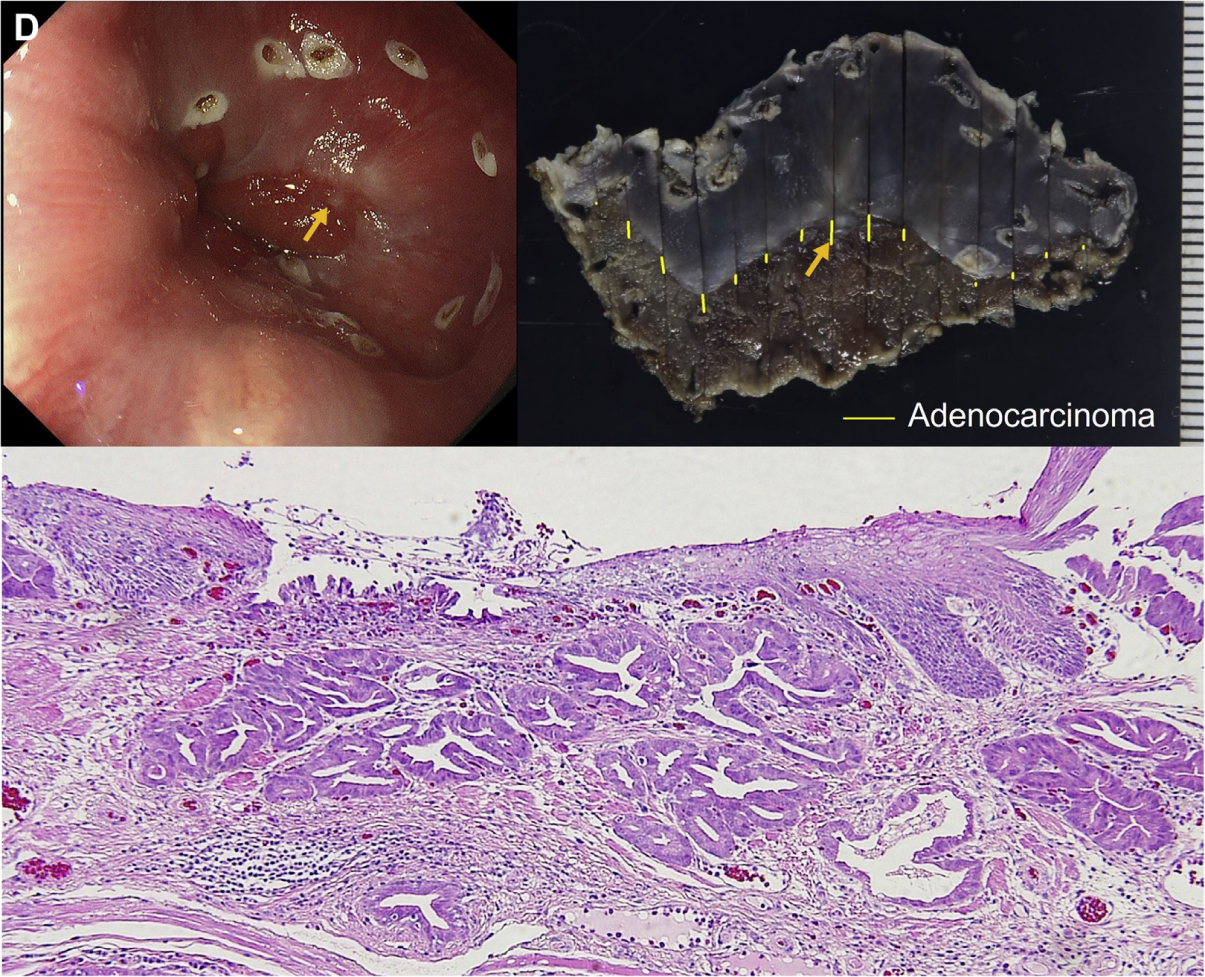# Differences in Squamous Epithelium Coverage of Barrett's Esophageal Adenocarcinoma Before, During, and After Antacid Use

**DOI:** 10.1016/j.gastha.2022.04.005

**Published:** 2022-04-14

**Authors:** Yohei Ikenoyama, Kyosuke Tanaka

**Affiliations:** 1 Department of Gastroenterology, Mie University Graduate School of Medicine, Tsu, Japan; 2 Department of Endoscopy, Mie University Hospital, Tsu, Japan

An 89-year-old man underwent esophagogastroduodenoscopy. Endoscopy revealed a depressed lesion partially covered with squamous epithelium (SE) on the squamocolumnar junction in Barrett’s esophagus (Figure A, arrow). Earlier, pathologic results of biopsies showed changes indefinite for neoplasia. Vonoprazan, a potassium-competitive acid blocker (P-CAB), was administered for 2 months, and the patient was re-examined. An endoscopy revealed SE covering a wide area, and the lesion became more difficult to recognize (Figure B). Pathologically, a biopsy specimen showed changes indefinite for dysplasia. Hence, vonoprazan administration was discontinued.

After 4 months, third endoscopy showed SE covering receded to the oral side, and a portion of the lesion was exposed (Figure C). Biopsy of the lesion revealed well-differentiated adenocarcinoma. Endoscopic submucosal dissection was performed, and histopathology of the resected specimen revealed Barrett’s esophageal adenocarcinoma (BEA) in contact with the SE (Figure D).

The use of proton pump inhibitors or P-CAB often masks BEA with SE and makes diagnosis difficult. However, it was unclear whether BEA, once covered with SE, would be re-exposed after discontinuing proton pump inhibitor/P-CAB. This is a valuable case because changes in the SE covering BEA before, during, and after taking P-CAB were observed. Hence, discontinuation of antacid could be helpful for the recognition of BEA.

**Figure:**
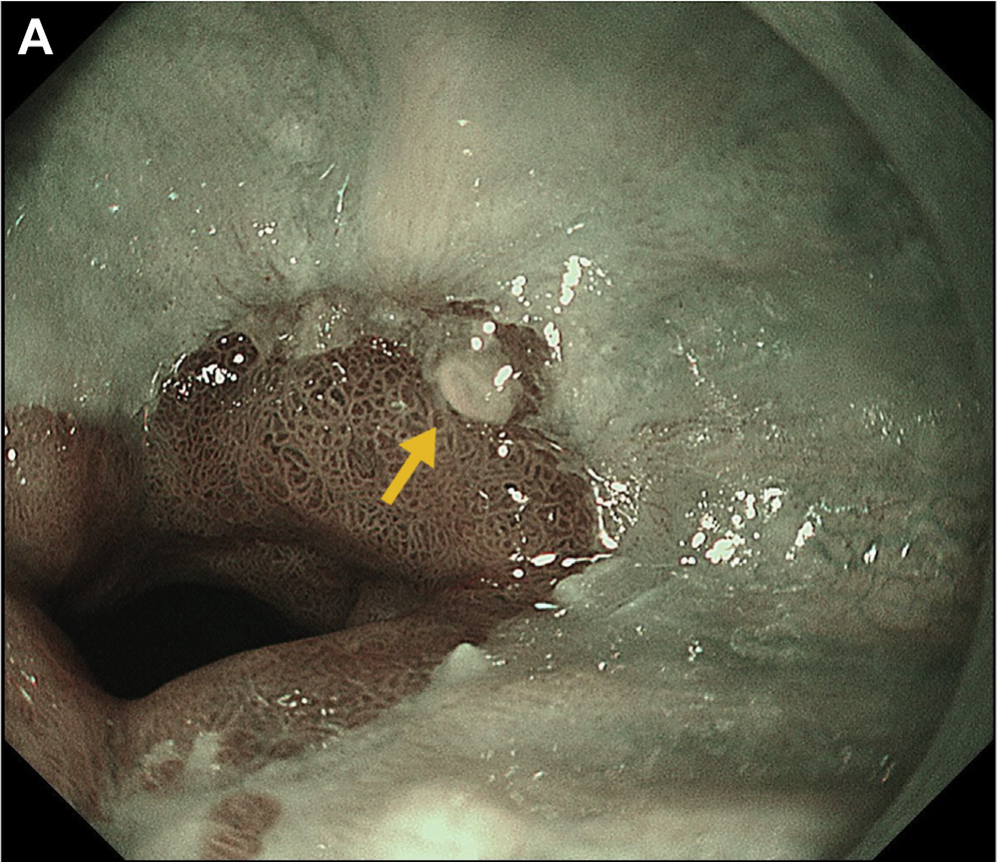

**Figure:**
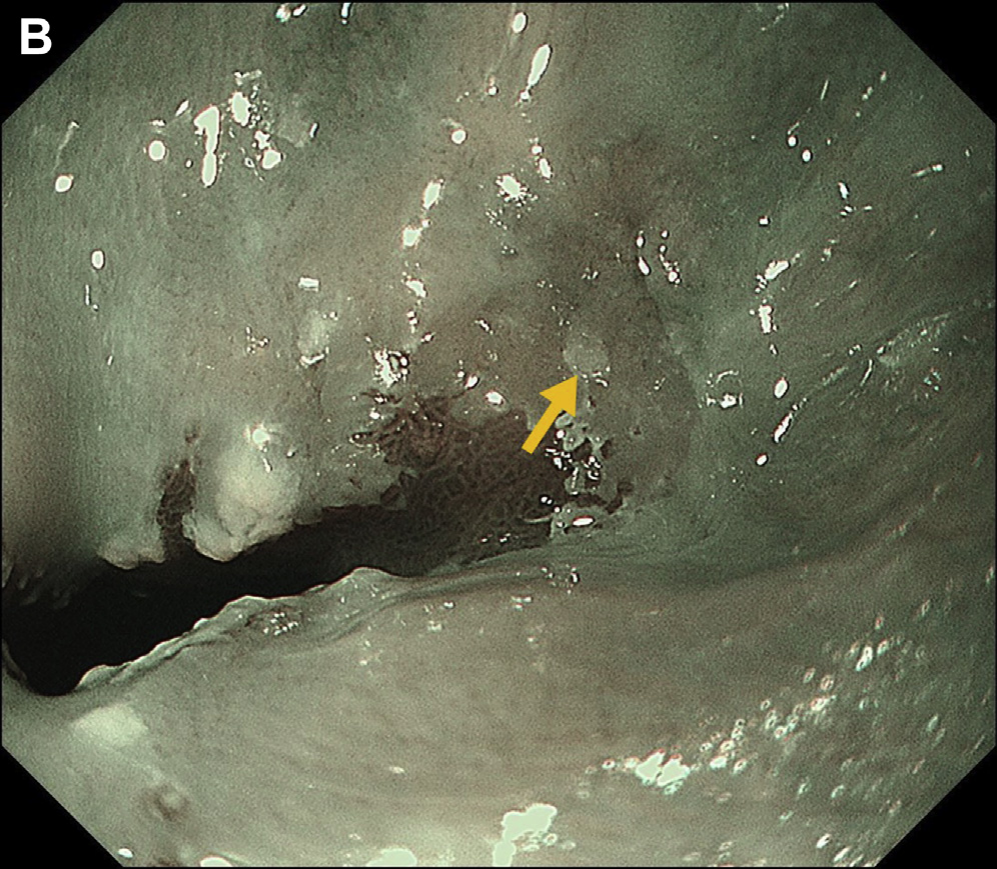

**Figure:**
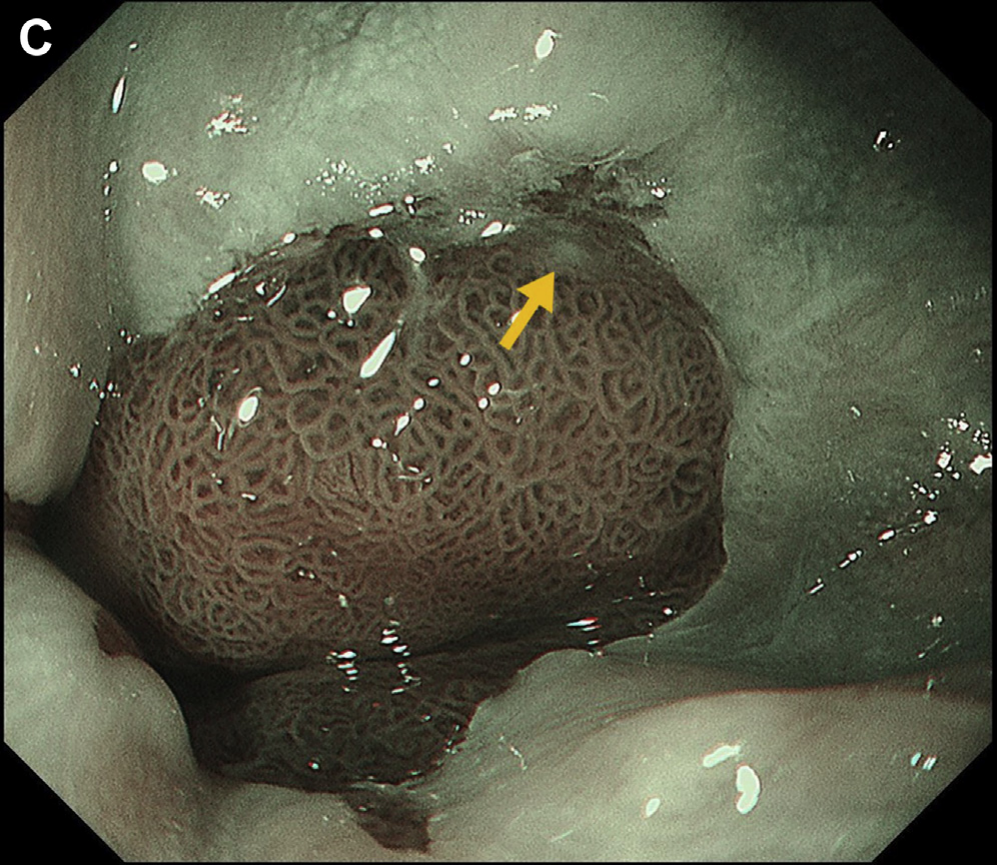

**Figure:**